# Cell stiffness predicts cancer cell sensitivity to ultrasound as a selective superficial cancer therapy

**DOI:** 10.1002/btm2.10226

**Published:** 2021-05-27

**Authors:** Eden Bergman, Riki Goldbart, Tamar Traitel, Eliz Amar‐Lewis, Jonathan Zorea, Ksenia Yegodayev, Irit Alon, Sanela Rankovic, Yuval Krieger, Itay Rousso, Moshe Elkabets, Joseph Kost

**Affiliations:** ^1^ Department of Chemical Engineering Ben‐Gurion University of the Negev Beer‐Sheva Israel; ^2^ The Shraga Segal Department of Microbiology, Immunology and Genetics, Faculty of Health Sciences Ben‐Gurion University of the Negev Beer‐Sheva Israel; ^3^ Institute of Pathology Barzilai University Medical Center Ashkelon Israel; ^4^ Department of Pathology, School of Health Sciences Ben‐Gurion University of the Negev Beer‐Sheba Israel; ^5^ Department of Physiology and Cell Biology Ben‐Gurion University of the Negev Beer‐Sheva Israel; ^6^ Department of Plastic Surgery and Burn Unit, Faculty of Health Sciences Soroka University Medical Center, Ben‐Gurion University of the Negev Beer‐Sheva Israel

**Keywords:** AFM measurements, mechanical properties of cancer cells, noninvasive therapy, selective cancer therapy, superficial cancer, ultrasound

## Abstract

We hypothesize that the biomechanical properties of cells can predict their viability, with Young's modulus representing the former and cell sensitivity to ultrasound representing the latter. Using atomic force microscopy, we show that the Young's modulus stiffness measure is significantly lower for superficial cancer cells (squamous cell carcinomas and melanoma) compared with noncancerous keratinocyte cells. In vitro findings reveal a significant difference between cancerous and noncancerous cell viability at the four ultrasound energy levels evaluated, with different cell lines exhibiting different sensitivities to the same ultrasound intensity. Young's modulus correlates with cell viability (*R* ^2^ = 0.93), indicating that this single biomechanical property can predict cell sensitivity to ultrasound treatment. In mice, repeated ultrasound treatment inhibits tumor growth without damaging healthy skin tissue. Histopathological tumor analysis indicates ultrasound‐induced focal necrosis at the treatment site. Our findings provide a strong rationale for developing ultrasound as a noninvasive selective treatment for superficial cancers.

## 1 INTRODUCTION

One of the most important developments in cancer biology over the past decade is the recognition that tumor growth, invasion, and metastasis are all intricately tied to the constituent cells' abilities to sense, process, and adapt to mechanical forces in their environment.^1^ An important part of the cancer progression process involves changes in the mechanical phenotype of the tumor cells and their microenvironment, as reflected by intrinsic changes in cell and tissue structure, mechanics, and the biophysical properties of the extracellular matrix.^2^, ^3^ For example, malignant cells are easier to deform compared with their noncancerous counterparts because their fewer, less organized F‐actin filaments produce a weaker cytoskeletal structure.^4^, ^5^, ^6^ Since malignant cells are more deformable, they may possess the ability to migrate through surrounding tissues more easily.^6^, ^7^

Squamous cell carcinoma of the head and neck (HNSCC)^8^, ^9^, ^10^, ^11^, ^12^ is an aggressive cancer, with patients reporting high levels of disease and treatment‐related symptoms affecting basic daily functions, such as speech, chewing and swallowing, and facial expressions. Currently, the most widespread and efficient treatment for superficial cancers is excisional surgery,^13^ although local irradiation and topical creams are also applicative.^14^ Patient recovery from these ablative procedures may be accompanied by lengthy wound healing processes and esthetic impairment. These unfavorable outcomes highlight the clinical need to develop an efficient treatment that does not harm normal cells and normal tissue function.

The use of ultrasound as a tool in cancer therapy has been studied since the 1940s^15^, ^16^ Studies from our laboratory^17^ and by others^18^, ^19^, ^20^, ^21^ have found that certain malignant cells are highly sensitive to ultrasonic irradiation. Ultrasound produces a variety of nonthermal mechanical bio‐effects,^22^, ^23^ inducing shear stress^24^, ^25^ in cells and stretch/compression distributions in the vicinity of the cellular surface through microstreaming around bubbles, cavitation, and acoustic streaming.^22^ Ultrasound pulsing reversibly perturbs the physical and subcellular structures of living cells.^26^ Consequently, transient membrane permeabilization (sonoporation) or cell death, depending on the ultrasound conditions,^22^, ^23^, ^27^, ^28^ can occur. Moreover, in vivo results from Azagury et al.^17^ indicate that the direct application of low‐intensity ultrasound to sarcoma tumor reduced tumor growth and increased tumor lysis in mice. However, more widespread and detailed studies are required before low‐intensity ultrasound can be used in clinical applications. For superficial HNSCC, such as on the lips and nose,^29^, ^30^, ^31^, ^32^, ^33^ the applicability of ultrasound as a treatment modality is expected to be relatively simple, because the ultrasound would be applied topically, as such tumors on a superficial organ can be easily accessed.

Although considerable research has evaluated the role of ultrasound in cancer therapy, historical review^34^ and recent comprehensive review^35^ showed no effect of ultrasound at all. It is difficult to compare and draw any conclusions from the contradictive results of various investigators, since so many different ultrasound application protocols and tumor model systems have been used. These studies led to our research hypothesis that *a single parameter*, *representing the biomechanical properties of different cell types*, *can predict their sensitivity to ultrasound treatment*. To test this hypothesis, we used atomic force microscopy (AFM) to undertake indentation measurements on different types of superficial cancer cells and thereby examine their deformability, as represented by their Young's modulus, which is a measure of the stiffness of an elastic material.^36^ The question of whether the biomechanical characteristics of malignant cells are broadly similar across all tumor types remains unanswered. Consequently, we focused on superficial cancers, particularly on HNSCC, as a model to test our hypothesis.

Gaining knowledge and understanding of the selective sensitivity to ultrasound energy of cancerous cells having different biomechanical properties is of fundamental as well as practical interest. This knowledge, combined with one of the primary advantages of ultrasound treatment, namely its potential for localized noninvasive application, should provide a solid basis for future clinical studies into personalized selective superficial cancer therapy.

## 2 RESULTS

### 2.1 Young's modulus measurements of various superficial cancer cell types

**FIGURE 1:**
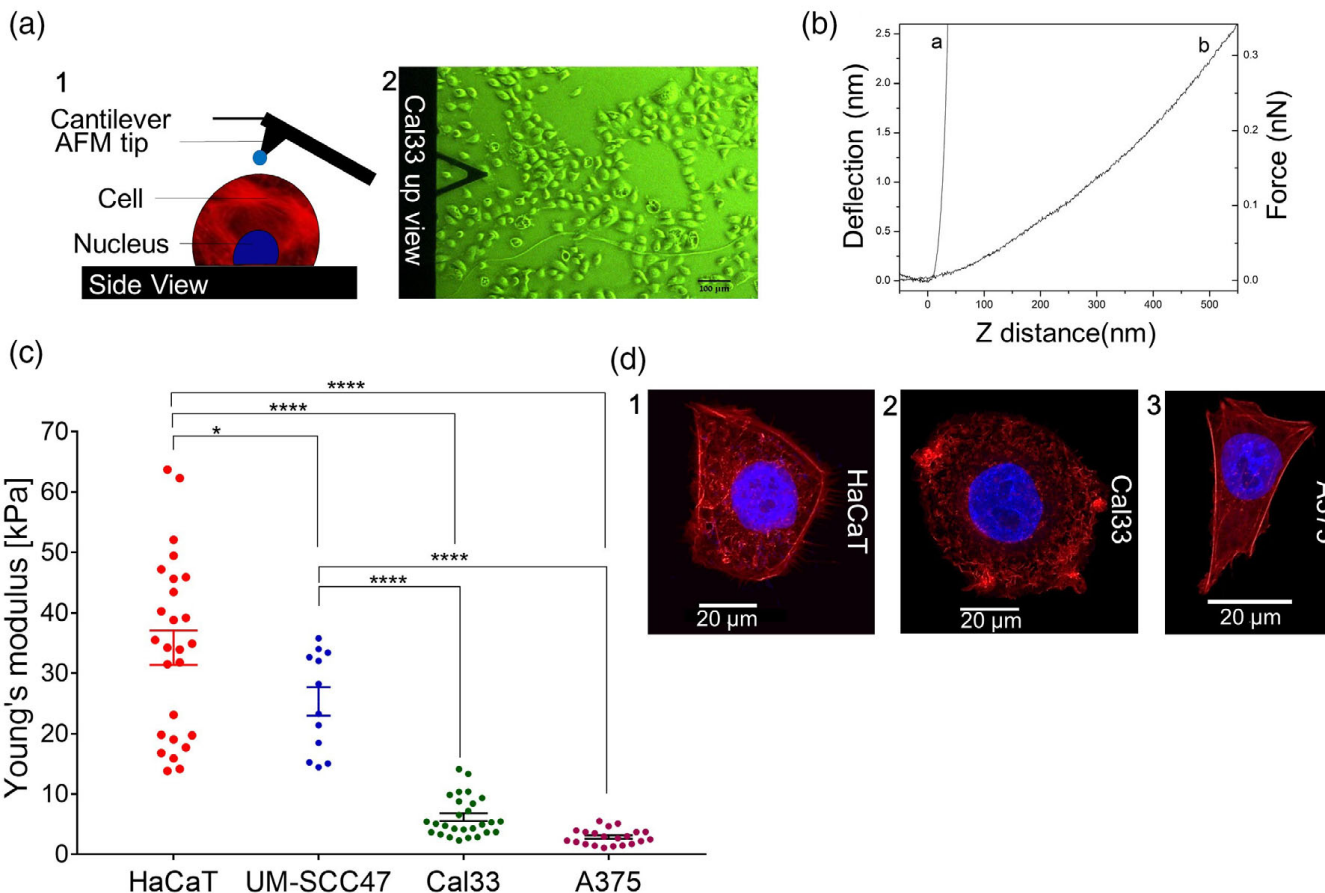
Cellular stiffness is associated with sensitivity to ultrasound treatment. (a) Atomic force microscopy (AFM) deflection measurement experimental set‐up: (1) Side view illustration of AFM deflection measurement. (2) Up view of the Cal33 cancer cell line during AFM measurement (optical microscope, bright‐field mode, ocular magnification 10×, objective magnification 10×, for total magnification 100×). (b) AFM analysis: A representative example of a deflection–force–distance plot for noncancerous HaCaT cells using MATLAB analysis based on the Hertz model: (Curve a) hard, nondeformable surface (glass); (Curve b) HaCaT cell. (c) Calculated Young's modulus values for different types of superficial cancerous (Cal33 and A375) and noncancerous (HaCaT) cells at 37°C. Error bars indicate *SEM*. Each dot is the mean of three measurements at different areas on the same cell (60 force–distance curves total). Statistical significance was calculated using one‐way ANOVA test, *p < 0.05, ****p < 0.0001. (d) Confocal images of different types of superficial cancerous and noncancerous cells with F‐actin labeled in red and the nucleus labeled in blue: (1) HaCaT (keratinocytes); (2) Cal33 (squamous cell carcinoma of the head and neck [HNSCC]); and (3) A375 (melanoma)

AFM was used to evaluate the biomechanical properties of cells from four different lines: noncancerous cells (HaCaT), HNSCC cells (UM‐SCC47 and Cal33), and melanoma cells (A375). Measuring the Young's modulus offers a means to quantify the mechanical differences between cells by measuring their deformability and plotting the resulting force–distance curves (Figure 1(a,b), respectively), with higher Young's modulus values indicating stiffer cells. As can be seen in Figure 1(c), the average Young's modulus of noncancerous HaCaT cells is 34 ± 3 kPa, which is significantly higher than the values for UM‐SCC47 (25 ± 2 kPa; *p* = 0.0295), Cal33 (6.2 ± 0.6 kPa; *p* < 0.0001), and A375 (1.6 ± 0.2 kPa; *p* < 0.0001).

The actin network, formed by actin filaments (F‐actin) or stress fibers, significantly contributes to the mechanical stability (elasticity or stiffness) of living cells,^7^, ^37^ and modifications to the actin cytoskeleton during the metastatic process correlate with cell malignancy^38^, ^39^, ^40^ The arrangement of fluorescently labeled F‐actin filaments in HaCaT, Cal33, and A375 cells was visualized by confocal fluorescence microscopy to verify whether the observed differences in their mechanical behaviors reflect differences in their F‐actin network structures. Figure 1(d) shows representative images from the examination of 73 cells, showing that the structures of the three cytoskeletons differ significantly from each other. HaCaT cells (*n* = 32; Figure 1(d1)) possess a pronounced network of red‐labeled actin filaments, which are localized in the peripheral region of the cell. By contrast, Cal33 (*n* = 28; Figure 1(d2)) and A‐375 (*n* = 13; Figure 1(d3)) cells possess fewer actin filaments, and the actin structures form a more disorganized and less cross‐linked network, which could contribute to their low Young's modulus values.

### 2.2 Correlation between cells' sensitivity to ultrasound and their Young's modulus

**FIGURE 2:**
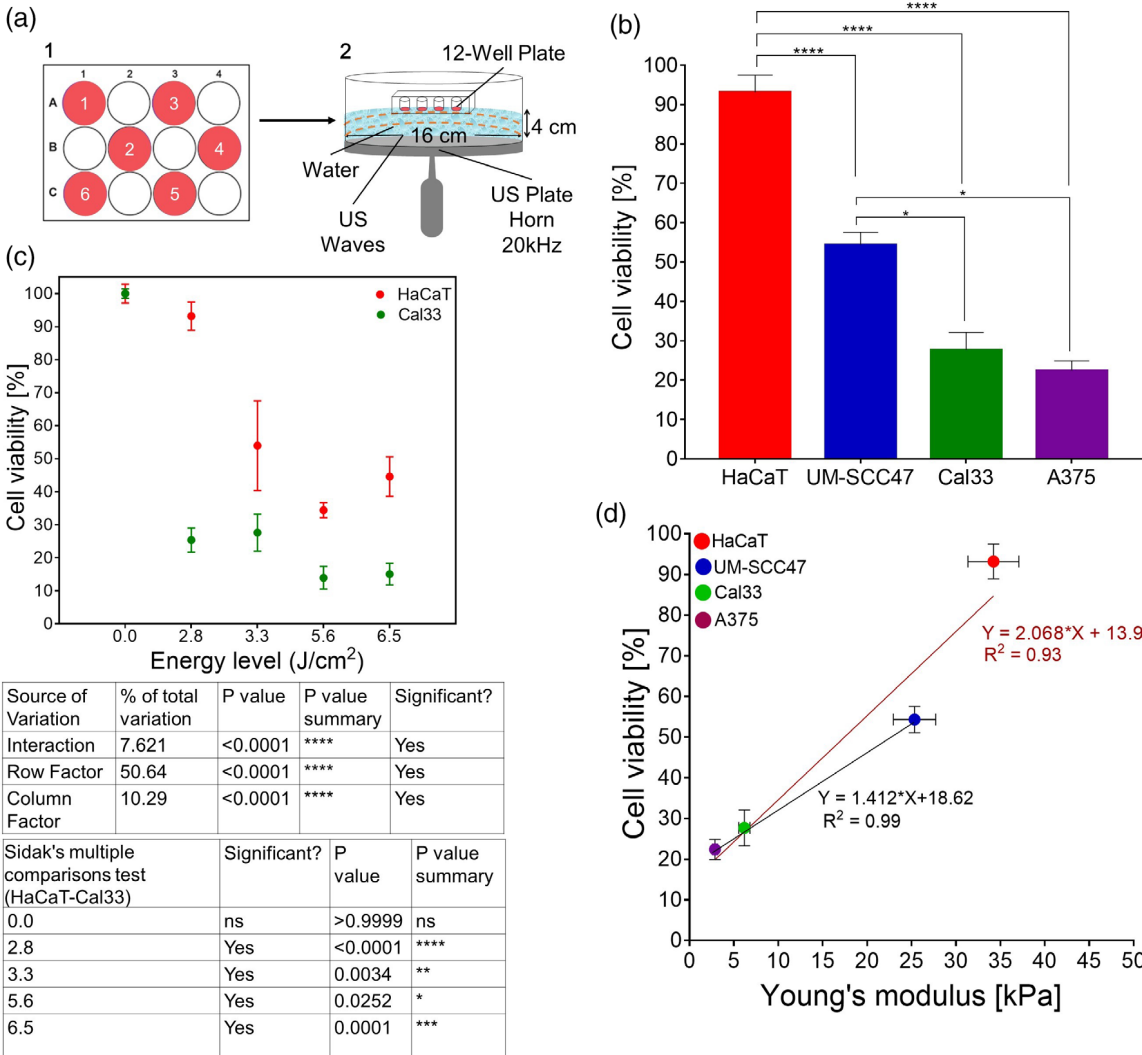
Effect of ultrasound exposure on cell viability in vitro. (a) Experimental setup: (1) Cell seeding in a 12‐well plate in a set order; (2) ultrasound plate horn set‐up (20 kHz). (b) Cell viability of noncancerous keratinocytes cells (HaCaT) compared with superficial squamous cell carcinoma of the head and neck (HNSCC) cells (Cal33) under ultrasound conditions of 0.139–0.164 W/cm^2^ intensity, 20 or 40 s application times, and a 50% duty cycle. The table shows the statistical significance calculated using two‐way ANOVA in terms of the energy level row factor, the cell viability column factor, and the interaction between them, where *p < 0.05, **p < 0.01, ***p < 0.001, ****p < 0.0001, and ns indicates a nonsignificant result. (c) Cell viability of various superficial cancer cell lines in vitro under ultrasound conditions of 0.139 W/cm^2^ intensity, 20 s application time, and a 50% duty cycle. Statistical significance was calculated using one‐way ANOVA test, with *p* values as per panel (b). (d) The correlation between cell viability and average Young's modulus for noncancerous and cancerous cells from various lines (red line) and for solely the cancerous cell lines (blue line) after their exposure to ultrasound under conditions of 0.139 W/cm^2^ intensity, 20 s application time, and 50% duty cycle

Having established the Young's modulus of the different cell types, we investigated whether it can predict cell sensitivity to ultrasound treatment. We exposed HNSCC cells (Cal33) and noncancerous cells (HaCaT) to different ultrasound operating conditions to identify the ultrasound parameters that cause damage to cancerous cells while being tolerated by healthy tissue. Figure 2(a) is a schematic presentation of our experimental setup for measuring cell viability following ultrasound exposure. Four ultrasound energy levels were tested in this experiment: 2.8, 3.3, 5.6, and 6.6 J/cm^2^. These energy levels were achieved using an ultrasound frequency of 20 kHz, intensities of 0.139 or 0.164 W/cm^2^, and exposure times of 20 or 40 s, while operating at a 50% duty cycle. As can be seen in Figure 2(b), there is a significant difference between the viabilities of the HaCaT and Cal33 cell lines at all the ultrasound energy levels evaluated (interaction *p* < 0.0001; row factor [energy level] *p* < 0.0001; column factor [cell viability] *p* < 0.0001). An ultrasound energy level of 2.8 J/cm^2^ did not affect the viability of HaCaT cells (~95 ± 4% remained viable), yet considerably decreased the viability of Cal33 cells (~27 ± 4% remained viable). Since higher ultrasound energy levels reduced noncancerous HaCaT cell viability (to ~50 ± 13%), we examined the effect of the 2.8 J/cm^2^ ultrasound energy level on the viability of other superficial cancer cell lines. Figure 2(c) presents the percentage of cells that remained viable for two additional tumor cell lines, UM‐SCC47 (HNSCC) and A375 (melanoma). It is important to note that, in addition to the different effects of ultrasound on noncancerous compared with cancerous cells, these results also demonstrate that various cancer cell types exhibit different sensitivities to the same ultrasound application.

The identification of a non‐molecular cellular parameter that differs between cancerous and noncancerous cells—in this case, Young's modulus, which is a biomechanical measure—potentially opens the way to personalized cancer therapy. Figure 2(d) (utilizing data from Figures 1(c) and 2(c)) represents cell viability as a function of Young's modulus at an ultrasound energy level of 2.8 J/cm^2^ for four different cell lines. The observed differences in cell viability following ultrasound application correlate with their stiffness, such that cells with a lower Young's modulus (less stiff, more elastic cells) are also less viable following ultrasound treatment. These results support our study hypothesis that a single biomechanical property can predict cell sensitivity to ultrasound treatment.

### 2.3 Ultrasound treatment delays tumor progression in vivo

To validate the potential of ultrasound as a treatment for superficial cancers in tumor‐bearing mice, we initially conducted a safety study to evaluate the effect of ultrasound on normal, healthy skin. Since we aimed to evaluate the effect of ultrasound in vivo, in which the ultrasound energy needs to permeate the ultrasound gel (coupling agent) placed above the skin surface and the dense tissue rather than an aqueous medium, the energy level applied for the in vivo experiments was two orders of magnitude higher than the energy level used in the in vitro experiments, mainly due to the large attenuation of ultrasound in the ultrasonic gel evident by the gel temperature increase requiring gel replacement every 30 s as described the materials section.

**FIGURE 3:**
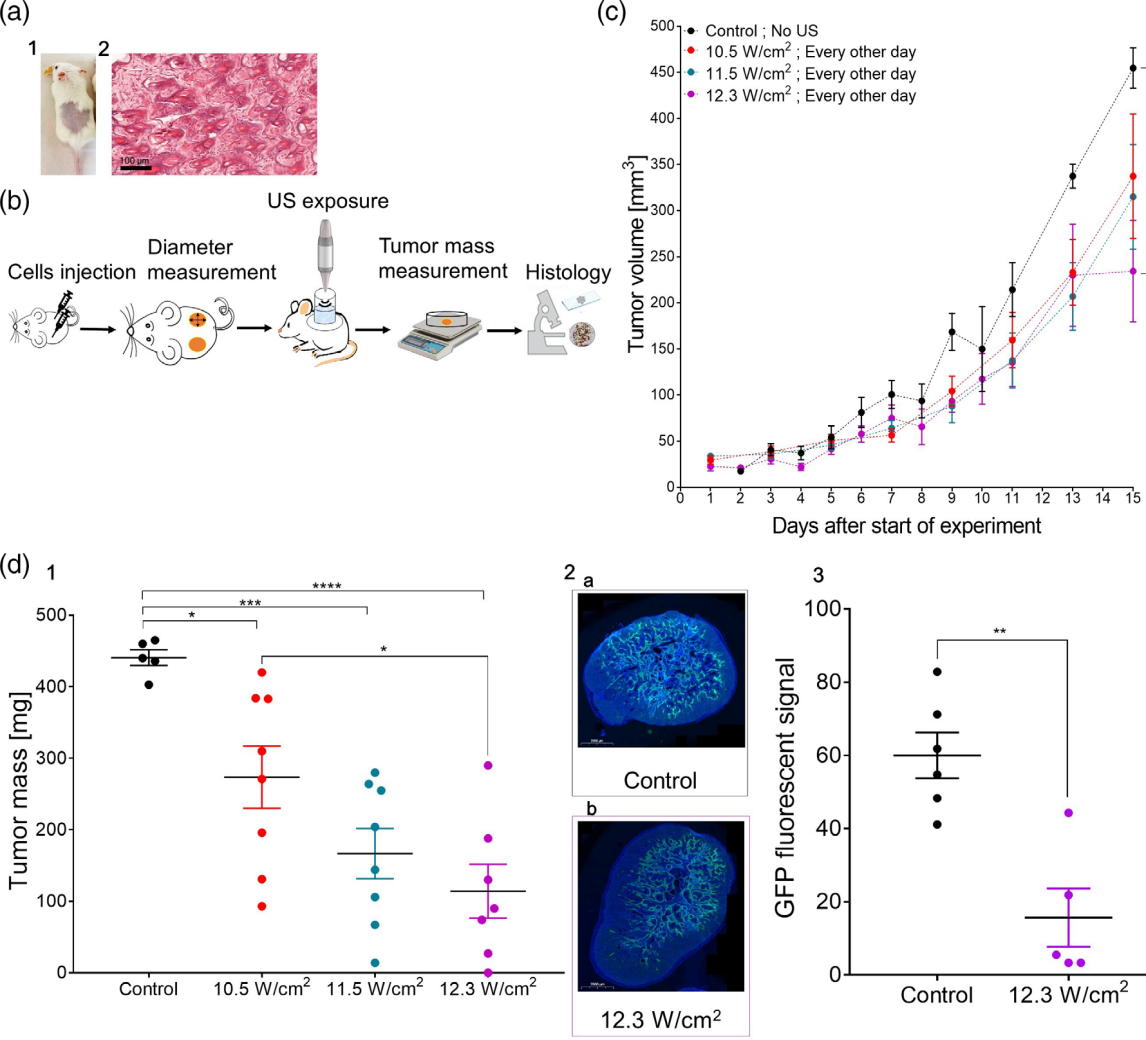
Ultrasound treatment delays tumor progression in vivo. (a) The effect of ultrasound on normal skin: (1) Visual view of NOD/SCID mouse skin after ultrasound exposure; (2) hematoxylin and eosin (H&E) histological analysis of mouse skin following exposure to ultrasound (12.3 W/cm^2^ intensity, 3 min application time, and a 50% duty cycle). (b) The in vivo procedure. (c) Effect on tumor volume of ultrasound treatment every other day over 15 days using three different ultrasound intensities for 1 min on a 50% duty cycle. (d) Effect of ultrasound intensity on squamous cell carcinoma of the head and neck (HNSCC) tumors. (1) Tumor mass measurements, 15 days after the treatment groups were first exposed to ultrasound, using three different intensities for 1 min on a 50% duty cycle. (2) Florescent scanning of Cal33‐green fluorescent protein (GFP) histological sections (GFP labeled green, nucleus labeled blue) for (a) the control group; (b) following ultrasound treatment at 12.3 W/cm^2^ every other day for 15 days. (3) GFP fluorescent signal analysis (using the ImageJ program) of the control group and a treatment group exposed to an ultrasound intensity of 12.3 W/cm^2^ after 15 days of treatment. Statistical significance was calculated using *t* test, **p < 0.01

We utilized an ultrasound application protocol that was previously tested in our lab^41^ and found safe for the skin of NOD/SCID mice, namely, operation for 3 min at an intensity of 12.3 W/cm^2^ and a 50% duty cycle (corresponding to an energy level of about 340 J/cm^2^). As can be seen in Figure 3(a), under these conditions, no external skin damage and no evidence of pathological abnormalities were observed.

For the efficacy study, the tumorigenic Cal33 cell line was injected under the skin of NOD/SCID mice. When the tumor reached 3–5 mm in diameter, three different ultrasound intensities were applied, 10.5 11.5, or 12.3 W/cm^2^, for 1 min on a 50% duty cycle every other day. Tumor diameter was measured for the calculation of its volume assuming an ellipsoid shape. The tumor mass was measured following its removal (see Figure 3(b) for experimental protocol). From treatment days 11–15, tumor growth was delayed in all three treatment groups compared with the untreated group (Figure 3(c)). On Day 15, the average tumor volume of the experimental group exposed to 12.3 W/cm^2^ was significantly (*p* = 0.0092) lower than that of the control group. Furthermore, there was a significant statistical reduction in tumor mass between all three experimental groups and the untreated groups (Figure 3(d1)). The smallest average tumor mass was found in group IV (Cal33 mice treated for 1 min every other day at an intensity of 12.3 W/cm^2^ and a 50% duty cycle), with one tumor entirely disappearing in this group. It is important to mention that none of the ultrasound treatments caused any visible damage to the exposed skin. Furthermore, reduced fluorescent signal was observed in the ultrasound treated tumors of Cal33‐green fluorescent protein (GFP) mice compared with control mice (Figures 3(d2) and 3(d3)). In Group IV, the area of the tumor comprised of cancer cells was reduced (15% ± 7%) compared with untreated control group (60% ± 6%). The tissue that did not express GFP may be either stromal cells or necrotic tumor cells. These results show that the reduction in tumor volume is proportional to the reduction in tumor mass.

**FIGURE 4:**
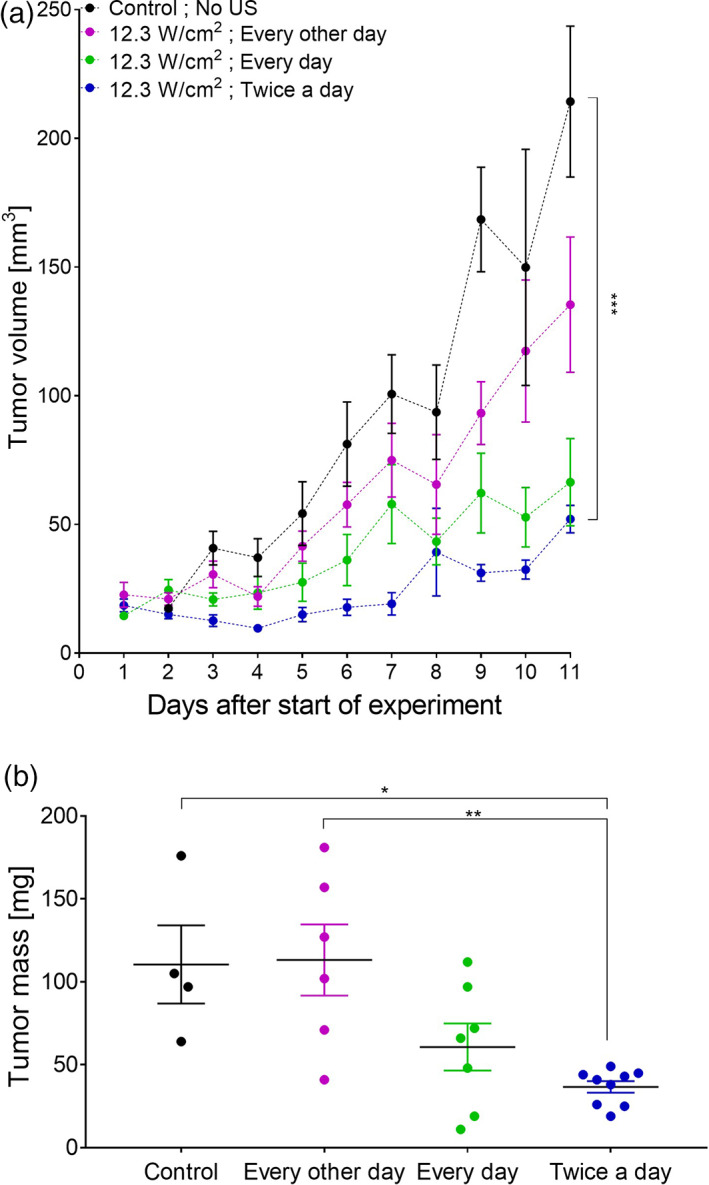
The effect of repeated ultrasound treatment on squamous cell carcinoma of the head and neck (HNSCC) tumor growth. (a) Tumor volume change in the control group (no ultrasound exposure) compared with groups treated with ultrasound (1 min operation time at 12.3 W/cm^2^ intensity and a 50% duty cycle) for 11 days on different treatment repetition schedules: ultrasound exposure every other day; once a day; or twice a day. (b) Tumor mass measurements 11 days after the treatment groups were first exposed to ultrasound. Statistical significance was calculated using one‐way ANOVA test *p < 0.05, **p < 0.01, ***p < 0.001, ****p < 0.0001

To further optimize the ultrasound treatment protocol to achieve the greatest tumor reduction in the shortest time under in vivo conditions, we examined various treatment repetition schedules to obtain the most effective treatment regime that could safely be administered to each tumor. We therefore examined tumor progression on Cal33 mice following ultrasound application at 12.3 W/cm^2^ for 1 min on a 50% duty cycle once every other day compared with once a day, and with twice a day treatments. The tumor volume growth kinetics (Figure 4(a)) indicate that the repetition of ultrasound treatment is associated with enhanced reduction in tumor volume (and consequently with reduced growth). The greatest differences in tumor volume (Figure 4(a)) and mass (Figure 4(b)) were obtained between the control group and the group exposed to ultrasound twice a day (Group VII).

During all the in vivo experiments, no abnormal behavior of the mice was observed throughout the treatment of 14 days. In addition, no effects were seen on the skin or abnormal mortality of the mice.

### 2.4 Ultrasound treatment‐induced necrosis in tumors

**FIGURE 5:**
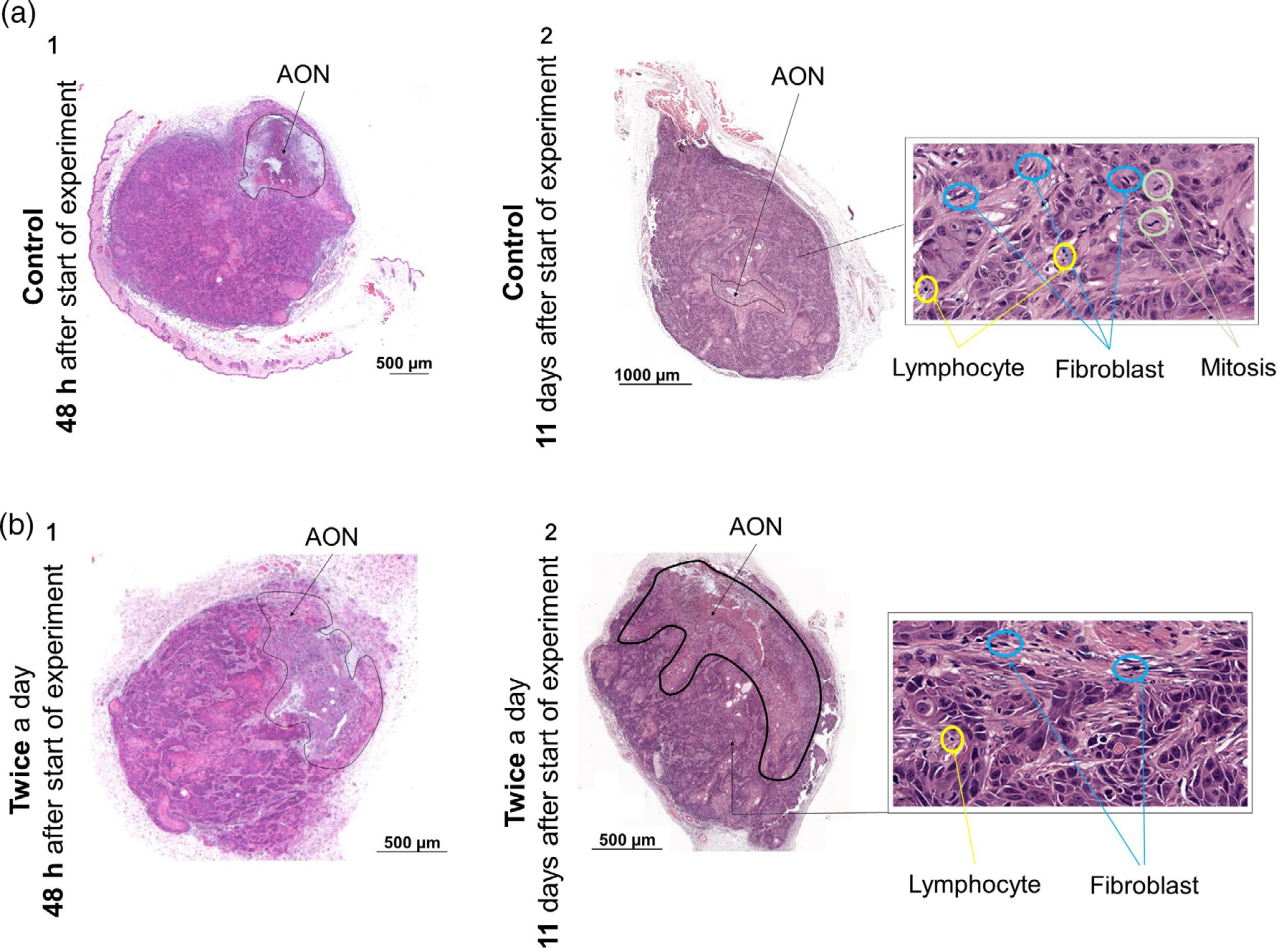
Representative images of hematoxylin and eosin (H&E) histological sections of Cal33 tumors and their morphological analysis. (a) Untreated and (b) treated tumor sections (1) 48 h and (2) 11 days after the first application of ultrasound to the treatment group. The necrotic area is indicated by a black outline. The treatment group received ultrasound treatment twice a day (1 min operation time at an intensity of 12.3 W/cm^2^ on a 50% duty cycle). Insets: ImageJ or CaseView images used for morphological analysis indicating the characteristics of necrosis

**FIGURE 6:**
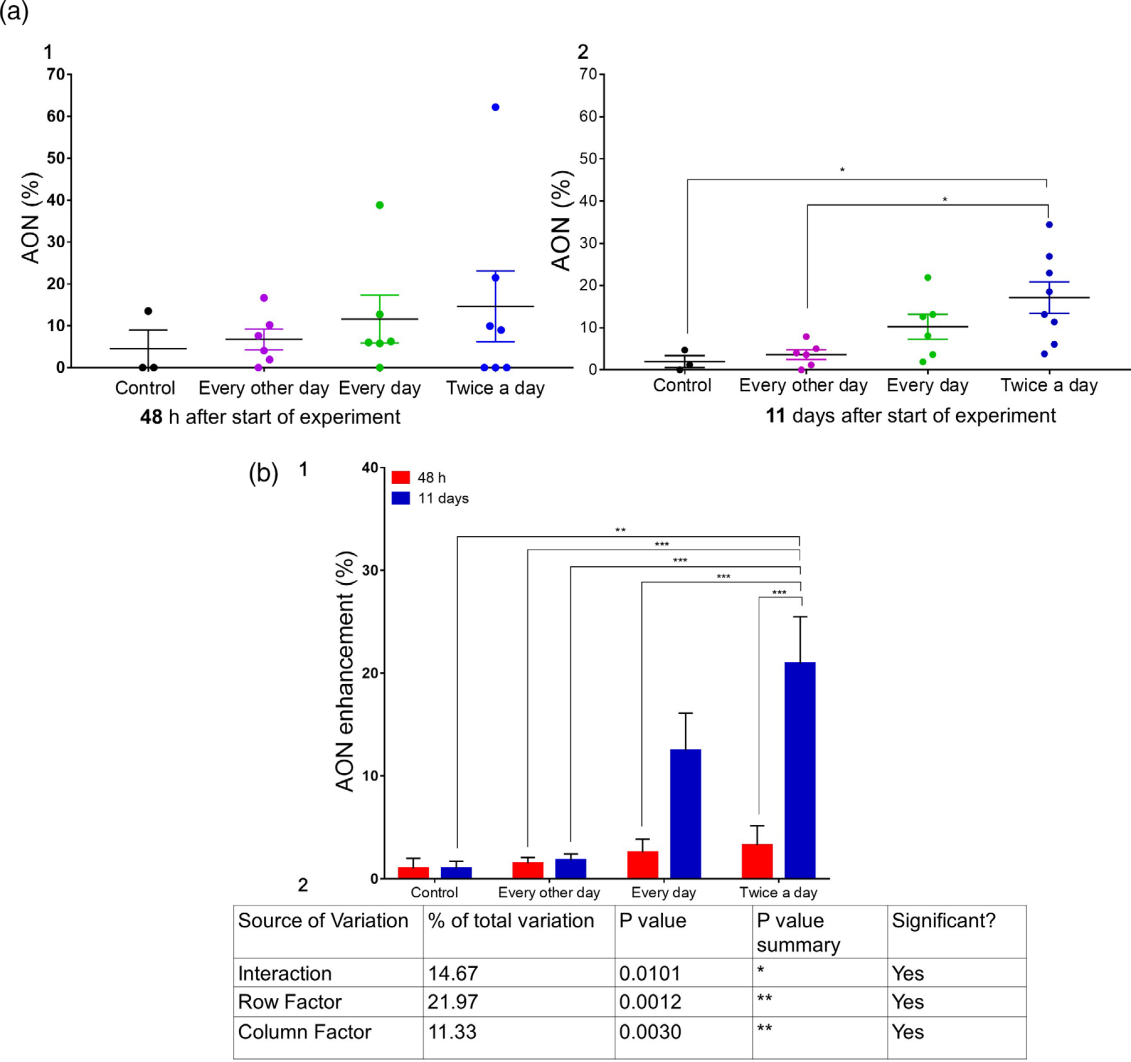
Ultrasound treatment‐induced necrosis in tumors: (a) Effect of ultrasound treatment (1 min operation time at 12.3 W/cm^2^ intensity and on a 50% duty cycle) on the necrotic area as a percentage of total tumor area (AON%) in groups treated according to different treatment repetition schedules compared with the control group, measured: (1) 48 h and (2) 11 days after first ultrasound application to the treatment groups. (b) (1) Tumor kinetics in the control group and in the treatment groups after 48 h (red) and 11 days (blue) of a twice a day ultrasound treatment schedule; and (2) two‐way ANOVA comparing the two treatment durations in terms of the energy level row factor, the cell viability column factor, and the interaction between them, where **p < 0.01, ***p < 0.001, ****p < 0.0001, and ns indicates a nonsignificant result

To understand the effect of ultrasound treatment (twice a day) on tumor mass and volume, a pathologist evaluated all the tumor cross sections 48 h and 11 days after the treatment groups were first exposed to ultrasound. The visual difference between the control and treatment groups was located in the area of necrosis (AON) (Figure 5). The AON is smaller for tumors from the control group compared with the treatment group, and the AON increases with increasing days of treatment. After 2 days of ultrasound treatment, there is no statistical difference between the control and treatment groups, whereas after 11 days of treatment the difference is statistically significant. Moreover, lymphocytes and fibroblasts cells are present in both the control and treatment groups, whereas atypical mitosis (indicating malignant tumor cells)^42^ is present only in the untreated control tumors. The largest value for mean AON as a percentage of tumor volume (AON%) was observed for the twice a day treatment group and the smallest was obtained in the control group (Figure 6(a1)). After 11 days, the difference between the twice a day treatment group and the control group was statistically significant (*p* = 0.0228) (Figure 6(a2)). Greater repetition of treatment yielded higher AON% values. After 11 days of treatment, AON% was 20 times greater for the twice a day treatment group compared with the control group, and this difference was statistically significant (*p* = 0.0032*)*. The AON% values for the grouped treated twice a day were also significantly greater than those of the group treated every other day (Figure 6(b)). All the histology results were consistent with the results of the in vivo experiments: 11 days of treatment administered twice a day at 12.3 W/cm^2^ produced the highest AON% and lowest tumor volume and mass.

## 3 DISCUSSION

Studies to evaluate ultrasound as a cancer treatment modality for superficial tumors have produced contradictory results, ranging from beneficial effects (mostly in studies performed on skin carcinomas) to no selective effect on tissues.^34^ Although the conflicting data may be attributed to the use of a wide variety of different experimental designs,^17^, ^20^ little consideration has been given to whether ultrasound treatment efficacy may also depend on the fundamental biomechanical properties of the target cells. Our analysis of superficial carcinoma cells shows that a single biomechanical parameter, namely, cell stiffness as quantified by Young's modulus by means of AFM indentation measurements, can predict the sensitivity of cancer cells to ultrasound treatment. This finding will enable the identification of additional cancers that are likely to be sensitive to ultrasound treatment. Furthermore, it widens the treatment modalities of relevance for a given cancer. Finally, it may serve as the basis for developing an ultrasound platform for the personalized, noninvasive therapy of superficial cancers by means of achieving the selective death of cancerous cells.

We used AFM to spatially map biomechanical properties across the surfaces of single cells and thereby obtain the mean Young's modulus values of a population of cells. Cells exhibit internal heterogeneity (for example, the nucleus is about 4–10 times stiffer than the cytoplasm^43^, ^44^, ^45^) and therefore we chose to perform the measurements using a probe having a spherical geometry to increase the contact area and decrease scattering. The appropriate model that corresponds to the sphere indenter is the modified Hertz model for living cells.^35^, ^46^

We found that highly aggressive cancer cells, such as melanoma cells (A375) and HNSCC cells (Cal33), had a lower Young's modulus than less aggressive cancer cells such as HNSCC cells (UM‐SCC47), with noncancerous cells (HaCaT) having the highest Young's modulus (Figure 1(c)). These results are consistent with those of other experimental AFM studies that presented the Young's modulus values of normal cells in comparison with those of cancer cells in different states of malignancy.^47^, ^48^, ^49^, ^50^, ^51^, ^52^ In addition, our study revealed a noticeable difference in the associated *SEM* between the four cell types, with the *SEM* values of the metastatic cancer cells being narrower than those associated with normal cells. These results, which have also been reported in the literature,^51^, ^53^ are probably due to differences in the cells' morphological characteristics, with tumor cells typically displaying anchorage‐independent growth patterns (i.e., cell rounding), whereas normal mesothelial cells exhibit a large, flat morphology.

Mechanistically, the association between Young's modulus and the organization and amount of F‐actin in cells has previously been defined.^7^, ^37^ Here, malignant cells expressed a less‐pronounced network of F‐actin filaments compared with non‐malignant cells (Figure 1(d)). Based on previous reports showing differences in stiffness between metastatic MDA‐MB‐231 and MCF‐7 breast cancer cells and non‐tumorigenic MCF‐10A cells,^5^, ^20^ we speculate that F‐actin, together with cell mechanics and behavior, explain selective cancer cell death following ultrasound treatment.

Generally, cell stiffness serves as a useful biomarker for the relative metastatic potential of ovarian and perhaps other types of cancer cells.^54^, ^55^ To the best of our knowledge, this is the first time that the cell stiffness parameter has been directly correlated with the efficacy of cancer treatment by ultrasound.

Cancerous cells (Cal33) were considerably more sensitive to ultrasound treatment than noncancerous cells (HaCaT) at all the ultrasound energy levels evaluated. Moreover, different cancer cells demonstrated different sensitivities to ultrasound at the same ultrasound energy level (Figure 2). The question of whether the measurement of Young's modulus by AFM can predict cell sensitivity to ultrasound has been answered in the affirmative by the excellent correlation (*R* ^2^ = 0.93 for noncancerous and cancerous cells on the same curve and *R* ^2^ = 0.99 when considering solely the three cancers examined) between Young's modulus and cell viability in vitro.

Subsequent in vivo experiments indicated that direct ultrasound, which induces mechanical stress on the cells,^24^, ^25^ results in the lysis of tumor cells and perturbation of the organization of the actin cytoskeleton.^56^ Exposure to ultrasound slowed tumor growth kinetics and caused focal necrotic damage to the cancerous tumor (Figure 5). Reductions in tumor mass and volume (Figure 4) and in AON (%) (Figure 6) correlated with treatment duration and repetition rate, suggesting that ultrasound has the potential to effectively treat superficial tumors.

Since ultrasound is known to cause cell death^20^, ^22^, ^23^, ^57^ via nonthermal mechanical effects,^58^, ^59^, ^60^ using low‐frequency ultrasound (20 kHz) is advantageous because it provides cavitation and acoustic streaming as a result of naturally dissolved gas bubble oscillation^61^ without the need for external intervention, such as cavitation nuclei or microbubbles. All these natural phenomena, which increase as the ultrasound energy level increases, can trigger biophysical effects, such as microstreaming, microjetting, and free‐radical formation, which may affect cell viability.^62^ The results show that the ultrasound penetrated the skin safely without causing damage to the healthy tissue and produced selective apoptosis of the cancerous cells only.

We suggest the difference in cancerous versus healthy cells structure and as a result their mechanical properties as presented by their modulus of elasticity, is the main cause for the selective difference in the effect caused by the shear stresses generated by ultrasound. Resulting in softer cells being more susceptible causing membranal rapture or porosity resulting in apoptosis.

Recent studies have revealed the potential of using ultrasound to activate an immune system response against cancer.^63^, ^64^, ^65^ One approach is to deliver immune stimulating agents to tumors by applying ultrasound to ultrasound‐sensitive carriers (e.g., tumor antigens or genes),^66^ whereas another approach aims to use the mechanical or thermal effects of ultrasound to enhance immune responses.^67^ These approaches endeavor to achieve immune modulation. The field of therapeutic immunomodulation is young and the mechanisms whereby ultrasound affects immune response are still not fully understood^57^ The current study was performed on mice lacking an immune system, and therefore the results do not reflect any effects of ultrasound exposure on the immune system.

Solid tumors are often first diagnosed by palpation, which may suggest that tumor tissues are more rigid that surrounding healthy tissues. Paradoxically, individual cancer cells are softer than their healthy counterparts.^68^ It follows that the correlation between Young's modulus and cell viability following ultrasound application may differ for tissue compared with cells. Nevertheless, our findings indicate that stiffness at the level of the individual cell is the key to selective ultrasound‐induced cell death.

Although we found a significant difference in tumor volume between the highest ultrasound intensity treatment group and the untreated control group and, in the treatment group, one of the tumors completely disappeared (Figure 3(d1)), the tumors continued to grow in both groups (Figures 3 and 4). This suggests that the treatment repetition schedules studied were not sufficient to eradicate the tumor and, therefore, additional work is necessary to optimize treatment for complete tumor eradication and the prevention of regrowth.

We showed that ultrasound treatment produced a quantitative effect on superficial tumor progression in vivo. These results, which are consistent with our previous report on breast cancer,^17^ suggest that cancer cell sensitivity to ultrasound may be related to a common phenomenon occurring in all cancer cells (such as reduced stiffness) regardless of their origin and type.

In view of the long‐term nature of cancer treatments, experiments comparing the mechanical properties of cells from cancers at different stages will prove useful to gain knowledge regarding the onset of superficial cancers and the ultrasound treatment conditions suitable for optimization of selective individual topical therapy. Moreover, investigating the mechanical properties of cancer cells may elucidate the physical mechanisms responsible for cancer metastasis. This can potentially lead to the development of novel strategies for cancer prevention and diagnosis. Overall, our findings suggest that the Young's modulus of superficial cancer cells can serve as a key parameter in the development of an ultrasound platform for personalized, noninvasive therapy that selectively kills cancerous cells without the need for cytotoxic drugs or ionizing radiation.

There are still challenges in the translational process to the clinic such as: the differences between the in vitro and in vivo mechanical properties of the cells, between the cells and in vivo tissues, and the natural variation in mechanical properties of healthy cells as a function of age and cell type.^69^, ^70^, ^71^, ^72^ We believe these could be addressed by the measurement of a tissues (cancerous and healthy) biopsy, instead of the cells or comparing the measurement of the cells from the biopsy, adjusting the ultrasound parameters based on the finding differences accordingly.

## 4 MATERIALS AND METHODS

### 4.1 Materials

Glycine (G7126), phosphate‐buffered saline (PBS; P4417), methyl sulfoxide‐*d*_6_ (547239), and trypan blue (T6146) were purchased from Sigma‐Aldrich (Israel). Acetone (01030521) and ethanol (05250502) were purchased from Bio‐Lab (Israel). Microscope slides (76 × 26 mm) were purchased from Thermo Scientific (Israel). Dulbecco's modified eagle medium (DMEM; 01‐055‐1A), Roswell Park Memorial Institute (RPMI) medium 1640 (01‐104‐1A), minimum essential medium (MEM; 01‐045‐1A), fetal bovine serum (FBS; 04‐121‐ 1A), glutamine (03‐020‐1B), trypsin (03‐052‐1A), trypan blue 0.5% (02‐102‐1B), and penicillin–streptomycin (03‐031‐1B) were purchased from Biological Industries (Israel). Presto Blue cell viability reagent (A13261) and Pro‐Long gold antifade reagent with 4′,6‐diamidino‐2‐phenylindole (DAPI; P36935) were purchased from Rhenium (Israel). Septol was purchased from Teva (Israel).

### 4.2 Cell lines and culture conditions

The human keratinocyte cell line (HaCaT) was grown in MEM and supplemented with glucose (4.5 mM), FBS (10% vol/vol), l‐glutamine (2 mM; 1% vol/vol), and penicillin–streptomycin (100 μg/ml penicillin and 100 μg/ml streptomycin; 1% vol/vol) in an incubator under a 5% CO_2_ atmosphere at 37°C. The cells were split every 2–3 days to prevent overpopulation as follows: the culture medium was removed from the flask and the cells were washed with filtered PBS. Cells were disconnected from the flask after the addition of 2 ml trypsin–EDTA and 10 min in an incubator. Following incubation, growth medium (10 ml) was added. The suspended cells were pipetted three to six times and divided into three flasks (4 ml each). Fresh medium was added to a total volume of 12 ml in each flask. The cells were returned to the incubator for 3 days for further proliferation.

The HNSCC cells lines Cal33 (human, tongue squamous cell carcinoma (SCC), Cal33‐GFP (human, tongue SCC, expressing GFP), and UM‐SCC47 (human, tongue SCC) were grown under conditions similar to those used for HaCaT, except that DMEM (rather than MEM) was used as the growth medium. For cell line A375 (human, malignant melanoma), the MEM growth medium was replaced by RPMI medium. All other experimental procedures were conducted identically for all cell lines.

### 4.3 Calculation of the Young's modulus of cells from AFM studies

Measurements were carried out with a JPK Nanowizard ultra‐speed AFM (Bruker, Berlin, Germany) mounted on an inverted optical microscope (Axio Observer; Carl Zeiss, Heidelberg, Germany). Borosilicate spherical AFM probes (diameter = ~2 μm; NovaScan) attached to triangular silicon cantilevers with a nominal spring constant of 0.1 N/m were used. The spring constant of the cantilever was determined experimentally by measuring its thermal fluctuations.^73^

Cells were seeded on 35 mm tissue culture dishes (TPP; 80,000 cells/ml). After 24 h, the growth medium was replaced, and cells were analyzed. To properly maintain the cells, a temperature of 37°C was maintained for the entire duration of the measurements using a microincubator perfusion chamber (PetryDishHeater, JPK instruments, Bruker), which holds a 35 mm cell culture dish attached to the microscope stage. Using an optical bright‐field microscope, isolated cells were selected for analysis to avoid possible influence of neighboring cells on the target cell's mechanical properties.

Cell stiffness was determined by indentation‐type experiments, as previously described.^51^, ^74^ Briefly, for each indentation measurement, a total of ~60 force–distance curves were acquired from three perinuclear locations on the cell surface at a scanning speed of 0.5–1 μm/s. The maximal applied loading force in each measurement was ~0.2–0.6 nN. Young's modulus was calculated by fitting a modified Hertz model^46^ to the force–distance curves. Repeated applications of force by this method have the potential to damage the sample irreversibly. Alternatively, the loading rate may affect the measured stiffness. Therefore, for each measurement consisting of ~60 force–distance curves, we plotted the measured point stiffness derived from each curve as a function of the measurement number and as a histogram. During each experiment, the measured stiffness values derived from the individual force–distance curves were found to distribute normally around a mean, which suggests that the cell did not undergo irreversible deformation during measurement. Data analysis was carried out using MATLAB software (The MathWorks, Natick, MA).

### 4.4 F‐actin confocal fluorescence imaging

For confocal fluorescence imaging, cells were grown and seeded (10,000 cells per 200 μl medium) in a μ‐slide eight‐well glass bottom plate. The medium was removed 24 h later and cells were fixed immediately with paraformaldehyde (PFA) in PBS (200 μl; 4% PFA). After 10 min of incubation at room temperature, the PFA was washed three times with 300 μl PBS. The cells were permeabilized for 2 min with 200 μl of 0.1% Triton X‐100 with 1:50 phalloidin in PBS. The filamentous actin (F‐actin) was labeled with phalloidin‐iFluor 555 reagent. After 10 min of incubation at room temperature, the well was twice washed with 300 μl PBS and then mounting medium with DAPI was added for nuclei staining. Fluorescence images were obtained using a confocal microscope (Zeiss LSM880 Airyscan).

### 4.5 In vitro cell viability assay

Cells were seeded at a density of 160,000 cells/ml in a 12‐well plate (each well contained 1 ml of culture medium). The culture medium was removed after 24 h of incubation and cells were washed with filtered PBS. Wells were filled with 100 μl Presto Blue (PB) reagent and 900 μl MEM and incubated in the incubator for 10 min. Then, a sample of 200 μl was drawn from each well of the 12‐well plate and was transferred into wells in a black 96‐well plate (three repetitions). The fluorescence in each well was measured by microplate reader spectrophotometer (Infinite M200, TECAN) at an excitation of 560 nm and emission 590 nm. The blank solution contained growth medium and PB (9:1 vol/vol).

### 4.6 Effect of ultrasound on cell viability in vitro

Cells were seeded at a density of 160,000 cells/ml in a 12‐well plate (each well contained 1 ml of culture medium) and their viability was tested using PB reagent, as described above. Afterward, the plate was washed with filtered PBS, filled with 1 ml of fresh medium and placed in a plate horn (QSONICA, 700 W, 20 kHz, 16 cm diameter) transducer container filled to a height of 4 cm with degassed water. All plates were placed identically to assure precisely the same position for efficient repetitions of the experiments.

For all experiments, ultrasound was applied at intensities of 0.139–0.164 W/cm^2^, for 20 or 40 s on a 50% duty cycle. After ultrasound exposure, the plates were incubated for 1 h at 37°C in a 5% CO_2_ atmosphere. Following ultrasound exposure, the same PB live cell viability procedure was performed. Cell viability was calculated as the number of treated cells viable after ultrasound exposure expressed as a percentage of the number of viable cells in the untreated sample, which were regarded as 100% viable.

### 4.7 Effect of ultrasound on tumor reduction: in vivo efficacy studies

Ultrasound treatment was carried out as previously described by Azagury et al.^17^ The current study (IL‐80‐12‐2015) was approved by the Institutional Review Board for animal welfare. Briefly, NOD/SCID mice aged 6 weeks old were injected subcutaneously with 100 μl of 1 × 10^6^ Cal33 HNSCC cell line/100 μl of PBS at two points on their backs. The ultrasound treatments started when tumors reached 3–5 mm in diameter (about 1 week after the injection), as measured manually by a caliper. Tumors that did not reach the appropriate size were not taken in account.

For ultrasound treatment, a cylindrical glass chamber (1.6 cm diameter) was placed over the tumor on the back of each anesthetized mouse and filled with ultrasound gel (3 ml at a temperature of ~4°C). The ultrasound probe was positioned 1 cm from the surface of the skin without touching the chamber walls. The ultrasound (QSONICA, 700 W, 20 kHz) was operated in an intensity range of 10.5–12.3 W/cm^2^ for 1–3 min on a 50% duty cycle using a probe with diameter 1.3 cm. Mice were anesthetized by injection of 100 mg/kg ketamine and 10 mg/kg xylazine before application of ultrasound. Groups that were exposed to the ultrasound more than once per day, requiring a total longer anesthesia per day, were connected to an isoflurane anesthetic system (SomnoSuite, low‐flow anesthesia system, from Kent Scientific Corporation) throughout the second sonication procedure. To minimize thermal effects, the ultrasonic gel was replaced with fresh gel every 30 s. During the procedure, before ultrasound application, the gel was kept inside an ice water bowl. After the ultrasound was turned off, the skin was cleaned with Septol.

For the safety experiments, healthy 6‐week‐old NOD/SCID mice (*n* = 2) were treated with ultrasound at an intensity of 12.3 W/cm^2^ for 3 min on a 50% duty cycle. Immediately after treatment, samples of the exposed skin were taken for histology examination.

To evaluate the effect of ultrasound on tumor reduction, different ultrasound intensities (10.5, 11.5, and 12.3 W/cm^2^) and treatment repetition rates (every other day, every day, and twice a day), were applied for 1 min on a 50% duty cycle. Cal33 mice (*n* = 43) were randomized into groups: (I) untreated (control) (*n* = 9); treatment every other day at (II) 10.5 W/cm^2^ (*n* = 4), (III) 11.5 W/cm^2^ (*n* = 4), or (IV) 12.3 W/cm^2^ (*n* = 10); (V) treatment every day at 12.3 W/cm^2^ (*n* = 7); and (VI) treatment twice a day at 12.3 W/cm^2^ (*n* = 9). During the experiments, tumor width and length (diameters) were measured manually using a caliper. Tumor volume was calculated using the ellipsoid volume equation under the assumption that the depth of the tumor is equal to the smaller diameter value. After 2 days, three mice from groups I, IV, and V, and four mice from group VI, were sacrificed. After 11 days, three mice from groups I and IV, four mice from group V, and five mice from group VI were sacrificed. After 15 days of treatment, three mice from group I, and four mice from groups II, III, and IV were sacrificed. The tumors were removed and washed with PBS. All the tumors were weighed (except for the tumors that were taken after 2 days for necrosis analysis) and transferred into 4% (wt/vol) PFA in PBS for 1 h/1 mm^3^ of tumor volume. Afterward, all the tumors were transferred into 70% ethanol until histology analysis was performed.

### 4.8 Histology

For histopathological preparation 4% (wt/vol) formalin‐fixed paraffin‐embedded HNSCC tumors were cut to 4 μm sections, mounted on microscope glass slides, and heated overnight at 65°C in a drying oven. Following dehydration, slides were stained with hematoxylin and eosin (H&E), scanned by a Panoramic MIDI II scanner (3D Histech) and analyzed by a pathologist. Necrotic areas within treated tumors were morphologically evaluated. First, the AON was marked and was calculated in arbitrary units using the ImageJ and CaseViewer programs, after which the AON was calculated as a percentage of the entire tumor volume (AON%). Morphological characteristics of necrosis consisted of areas of atypical mitosis, lymphocytes, fibrin, acute inflammation, and tissue loss. Results are presented in AON%. Statistical analysis was carried out by GraphPad Prism 7.03 software, significance set at *p* = 0.05.

### 4.9 Statistical analysis

Statistical analysis was performed using GraphPad Prism 7.03 software, presented as mean ± *SEM*. All cellular experiments were repeated at least three times. For experiments involving less than two groups, one‐way ANOVA was used. For experiments involving two groups, a two‐tailed Student's unpaired *t* test was performed to compare the control versus treatment groups. For experiments involving more than two groups, two‐way ANOVA was used. Values of *p* ≤ 0.05 were considered significant. For pathological analysis, H&E images were analyzed by Panoramic Viewer Histoquant software (3D Histech), and a one‐way ANOVA test was performed to compare control vs. treatment groups.

## CONFLICT OF INTERESTS

J. K. is an inventor on a U.S. patent application 14/198,701 on low intensity ultrasound therapy of hyperproliferative diseases and disorders. The authors declare no other conflict of interests.

## AUTHOR CONTRIBUTIONS

**Riki Goldbart**: Conceptualization; formal analysis; investigation; methodology; supervision; validation; writing‐original draft; writing‐review and editing. **Tamar Traitel**: Conceptualization; formal analysis; investigation; methodology; supervision; validation; writing‐original draft; writing‐review and editing. **Eliz Amar‐Lewis**: Methodology; writing‐review and editing. **Jonathan Zorea**: Investigation; methodology. **Ksenia Yegodayev**: Investigation; methodology. **Irit Alon**: Formal analysis; writing‐original draft. **Sanela Rankovic**: Formal analysis; investigation; methodology; software; writing‐original draft. **Yuval Krieger**: Conceptualization; writing‐original draft. **Itay Rousso**: Conceptualization; data curation; formal analysis; methodology; project administration; resources; supervision; validation; writing‐original draft; writing‐review and editing. **Moshe Elkabets**: Conceptualization; data curation; formal analysis; funding acquisition; methodology; project administration; supervision; validation; writing‐original draft; writing‐review and editing. **Joseph Kost**: Conceptualization; data curation; formal analysis; funding acquisition; methodology; project administration; supervision; validation; writing‐original draft; writing‐review and editing.

5

### PEER REVIEW

The peer review history for this article is available at https://publons.com/publon/10.1002/btm2.10226.
